# Modified Polyethylene Oxide Solid-State Electrolytes with Poly(vinylidene fluoride-hexafluoropropylene)

**DOI:** 10.3390/molecules30112422

**Published:** 2025-05-31

**Authors:** Jinwei Yan, Wen Huang, Tangqi Hu, Hai Huang, Chengwei Zhu, Zhijie Chen, Xiaohong Fan, Qihui Wu, Yi Li

**Affiliations:** 1 Xiamen Key Laboratory of Marine Corrosion and Smart Protective Materials, Cleaning Combustion and Energy Utilization Research Center of Fujian Province, Key Laboratory of Energy Cleaning Utilization, Development, College of Marine Equipment and Mechanical Engineering, Jimei University, Xiamen 361021, China; yanjinwei2001@163.com (J.Y.); 15159303587@163.com (H.H.); 15798082139@163.com (C.Z.); rockljj@jmu.edu.cn (Z.C.); echo_fan@jmu.edu.cn (X.F.); 2 Jiangsu Key Laboratory of Advanced Functional Polymer Materials, Department of Polymer Science and Engineering, College of Chemistry, Chemical Engineering and Materials Science, Soochow University, Suzhou 215123, China; 20234209214@stu.suda.edu.cn (W.H.); 20245209003@stu.suda.edu.cn (T.H.)

**Keywords:** PEO, P(VDF-HFP), solid-state electrolyte, lithium-ion batteries

## Abstract

Lithium-ion batteries are restricted in development due to safety issues such as poor chemical stability and flammability of organic liquid electrolytes. Replacing liquid electrolytes with solid ones is crucial for improving battery safety and performance. This study aims to enhance the performance of polyethylene oxide (PEO)-based polymer via blending with poly(vinylidene fluoride-hexafluoropropylene) (P(VDF-HFP)). The experimental results showed that the addition of P(VDF-HFP) disrupted the crystalline regions of PEO by increasing the amorphous domains, thus improving lithium-ion migration capability. The electrolyte membrane with 30 wt% P(VDF-HFP) and 70 wt% PEO exhibited the highest ionic conductivity, widest electrochemical window, and enhanced thermal stability, as well as a high lithium-ion transference number (0.45). The cells assembled with this membrane electrolyte demonstrated an excellent rate of performance and cycling stability, retaining specific capacities of 122.39 mAh g^−1^ after 200 cycles at 0.5C, and 112.77 mAh g^−1^ after 200 cycles at 1C and 25 °C. The full cell assembled with LiFePO_4_ as the positive electrode exhibits excellent rate performance and good cycling stability, indicating that prepared solid electrolytes have great potential applications in lithium batteries.

## 1. Introduction

With the improvement of people’s living standards, the overexploitation of energy resources has led to the gradual depletion of nonrenewable energy sources, which results in the development and utilization of green energy. However, the instability and intermittency characteristics of green energies (e.g., solar energy, wind energy, and tidal energy) demand continuous optimization of energy storage devices and technologies [1,2,3]. Among various energy storage devices, rechargeable lithium-ion batteries (LIBs) have emerged as the most attractive electrochemical storage systems due to their high energy density [4,5], high power [6,7], and long cycle life [8]. LIBs typically utilize carbon materials such as graphite as the anode and various lithium-containing active oxides as the cathode. In the view of application in electric vehicles, further enhancement in energy density and safety of LIBs remains the core objective of current research. To address the current technological bottlenecks, extensive research has been conducted by researchers attempting to replace graphite anodes with lithium metal anodes [9,10]. Compared with graphite anodes, lithium metal anode offers a theoretical specific capacity of 3860 mAh g^−1^, which is dozens of times higher than 372 mAh g^−1^ of graphite. Additionally, the use of lithium metal anodes can significantly increase the cell potential, thereby enhancing the energy density [11,12,13]. However, the commercialization of lithium metal anodes faces two critical safety challenges: the formation and propagation of lithium dendrites during charging and the flammability of conventional organic electrolytes. Notably, lithium dendrites can penetrate the separator, causing short circuits and subsequent battery failure [14,15]. Therefore, the adoption of solid electrolytes to replace liquid ones has emerged as an effective strategy to suppress lithium dendrite growth and enhance safety [16,17].

Solid electrolytes can be generally divided into two types: inorganic and polymer solid electrolytes. In contrast to inorganic solid electrolytes, polymers come with advantages such as straightforward synthesis, low cost, and high mechanical toughness. As a result, they have been widely employed in solid-state LIBs [18,19]. The most commonly used polymer electrolytes include polyethylene oxide (PEO) [20], polyacrylonitrile (PAN) [21], and polyvinylidene fluoride (PVDF) [22]. However, polymer solid-state electrolytes still face significant challenges in practical applications, such as low ionic conductivity resulting from their high crystallinity and poor mechanical properties that fail to suppress lithium dendrite growth effectively [23]. To address these challenges, extensive research has been conducted. For example, Wu et al. designed a MOF-derived nanoporous multifunctional filler, which was uniformly dispersed in a PEO matrix [24]. This dispersion reduced PEO crystallinity, enabling the assembled battery to exhibit superior ionic conductivity and excellent cycling stability at room temperature. Liang and his colleagues incorporated hydroxyapatite (HAP) as a filler into a PEO-based solid-state electrolyte [25]. This filler was evenly distributed within the PEO matrix, which hindered the crystallization of PEO and thus boosted the ionic conductivity. The battery assembled with this electrolyte showed outstanding electrochemical performance at room temperature. Li and colleagues prepared a composite electrolyte (PEO/LiTFSI/MnO_2_) by incorporating MnO_2_ nanosheets into the PEO/LiTFSI system [26]. Han and colleagues improved ionic conductivity by incorporating PEO with magnetically functionalized sepiolite nanowires (KFSEP), where the aligned nanowires provide ion-conductive pathways. The assembled batteries exhibit excellent electrochemical performance at elevated temperatures [27]. Li et al. used TDI (hexamethylene triisocyanate) as a “bridge” to connect PEO (poly(ethylene oxide)) molecular chains and SiO_2_ (silica) nanoparticles, forming an organic–inorganic network structure. In this structure, the TDI-SiO_2_ nanoparticles effectively inhibit the crystallization of PEO, thereby enhancing ionic conductivity. The assembled batteries exhibit high ionic conductivity and a wide electrochemical window at elevated temperatures [28]. The addition of MnO_2_ nanosheets lessened the crystallinity of PEO and consequently enhanced its ionic conductivity. As a result, the battery assembled with this electrolyte showed good capacity output at room temperature. Incorporating suitable fillers into the PEO-based polymer electrolytes has been demonstrated as an effective strategy to address the critical challenges of low ionic conductivity and lithium dendrite growth suppression.

In the current study, we prepare polymer solid-state electrolytes via blending PEO with polyvinylidene fluoride-hexafluoropropylene (P(VDF-HFP)) at different ratios. The addition of P(VDF-HFP) disrupts the chain regularity of PEO, thereby reducing its crystallinity. Moreover, the strong electron-withdrawing C-F groups in P(VDF-HFP) facilitate the dissociation of lithium salts, leading to improved ionic conductivity. Results showed that the polymer electrolyte prepared with 30 wt% P(VDF-HFP) and 70 wt% PEO exhibited superior electrochemical performance at room temperature. At 25 °C, the electrolyte achieved an ionic conductivity of 8.89 × 10^−3^ S cm^−1^. At a current density of 0.2C and 25 °C, the battery using this electrolyte delivered a reversible specific capacity of 159.82 mAh g^−1^. At 0.5C and 25 °C, the initial reversible specific capacity was 138.88 mAh g^−1^, retaining a relatively high capacity of 122.70 mAh g^−1^ after 200 cycles. Even at 1C and 25 °C, the reversible specific capacity remained 112.77 mAh g^−1^ after 200 cycles. Collectively, the blended PEO/P(VDF-HFP) electrolyte not only significantly enhances the ionic conductivity and cycling stability but also effectively suppresses the lithium dendrite growth, providing robust support for the safety and stability of LIBs. The full cell assembled with LiFePO_4_ as the positive electrode shows excellent rate performance and good cycle stability.

## 2. Results and Discussion

**Figure 1:**
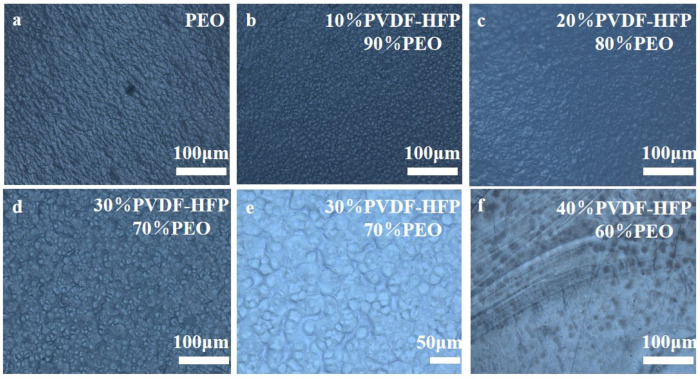
Optical electron microscopy images of the electrolyte membranes with different compositions: (**a**) PEO, (**b**) 10%P(VDF-HFP)/90%PEO, (**c**) 20%P(VDF-HFP)/80%PEO, (**d**) 30%P(VDF-HFP)/70%PEO, (**e**) 30%P(VDF-HFP)/70%PEO, (**f**) 40%P(VDF-HFP)/60%PEO.

Figure 1a–f presents the optical electron microscopy images of the prepared PEO polymer solid electrolyte and PEO/P(VDF-HFP) blends at various ratios. Figure 1a shows that the surface of pure PEO exhibits a long-chain structure, whereas the surface morphology evolves into a granular structure with the addition of P(VDF-HFP), as observed in Figure 1b–f. No phase separation occurred, confirming the good compatibility between the two blended polymers. However, the blend membranes with different P(VDF-HFP) contents consist of particles of various sizes. From Figure 1b–d, it can be seen that increasing the P(VDF-HFP) proportion leads to gradually larger particles and reduced compactness on the electrolyte membrane surface. However, when the P(VDF-HFP) content was 40%, the particles on the surface of the electrolyte membrane became more uneven and smaller in size. The blending of PEO and P(VDF-HFP) introduces intermolecular interactions between the two polymers, causing the formation of granular structures in the solid electrolyte membranes. These granules increase the amorphous regions, reduce crystallinity, and therefore, enhance the ionic conductivity. Moreover, the granular morphology improves the mechanical strength of the electrolyte, thereby effectively suppressing lithium dendrite growth [29].

**Figure 2:**
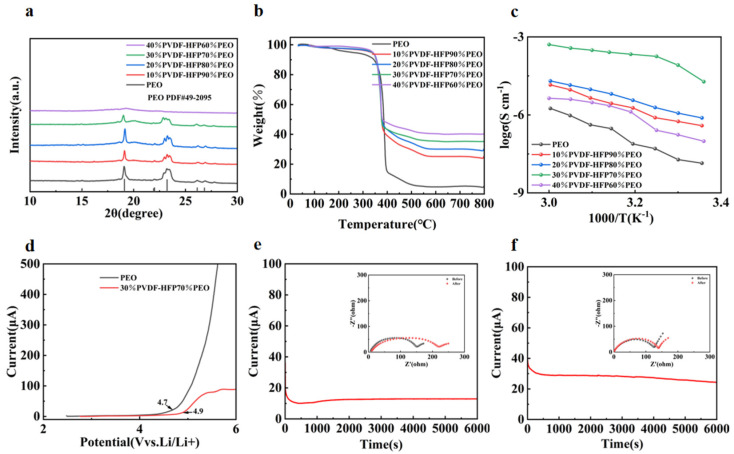
(**a**) XRD patterns of the electrolyte membranes with different compositions, (**b**) TGA curves of electrolyte membranes with different compositions under nitrogen atmosphere, (**c**) ionic conductivities of PEO and PEO/P(VDF-HFP) blends at various ratios across different temperatures, (**d**) LSV curves of PEO and 30%P(VDF-HFP)/70%PEO electrolytes, (**e**) i-t polarization curves of PEO electrolyte and symmetric cell impedance before/after polarization, (**f**) i-t polarization curves of 30%P(VDF-HFP)/70%PEO electrolyte and symmetric cell impedance before/after polarization.

Figure 2a displays the X-ray diffraction (XRD) patterns of pure PEO, 10%-P(VDF-HFP)/90%-PEO, 20%-P(VDF-HFP)/80%-PEO, 30%-P(VDF-HFP)/70%-PEO, and 40%-P(VDF-HFP)/60%-PEO electrolyte membranes. The pure PEO electrolyte exhibits strong characteristic crystalline peaks at 19° and 23°, which are also present in the XRD patterns of PEO/P(VDF-HFP) blends with varying compositions [30]. In the XRD spectra of the solid electrolyte membranes containing P(VDF-HFP), the peak intensity of PEO’s characteristics is attenuated, indicating that the addition of P(VDF-HFP) disrupts the crystalline phase of PEO and increases the amorphous regions [31]. The motion of lithium ions in PEO primarily relies on the vibrational migration of -C-O-C- segments, and this behavior is influenced by the degree of microstructural disorder in PEO [32,33]. The addition of P(VDF-HFP) disrupts the chain regularity of PEO, thereby exposing more ether groups that were previously confined within PEO crystalline regions and increasing the proportion of effectively available ether groups. The increased number of -C-O-C- segments promotes the dissociation of lithium salts, thereby enhancing ionic conductivity.

Figure 2b presents the TGA results of the prepared polymer solid electrolytes. When the temperature was first increased from 25 °C to 150 °C, the mass loss observed can be attributed to the physically adsorbed water and residual anhydrous acetonitrile in the electrolyte membranes. Within the temperature range of 150 °C to 350 °C, the pure PEO electrolyte exhibits significant mass loss, whereas the electrolytes incorporating P(VDF-HFP) show no noticeable mass reduction during this period. This discrepancy becomes more pronounced with increasing P(VDF-HFP) content in the electrolyte. However, when the temperature exceeded 400 °C, all five electrolyte membranes experienced a sharp mass decline due to the decomposition of PEO, P(VDF-HFP), and the LiTFSI at high temperatures. When the temperature approached 530 °C, no decomposable substances remained, and the thermogravimetric curve stabilized. The stable portion of the curve corresponds to residual carbonaceous residues in the pure PEO electrolyte. For the electrolytes containing P(VDF-HFP), the TGA curves stabilized after 630 °C, with the residual material consisting of carbonaceous residues and fluorinated residues from the PEO/P(VDF-HFP) blends. The TGA results demonstrate that the PEO/P(VDF-HFP) blended solid electrolytes exhibit superior thermal stability at higher temperatures, thereby enhancing the safety performance of the entire battery.

Electrochemical impedance spectroscopy (EIS) measurements were conducted on SS symmetric cells assembled with electrolyte membranes of different compositions at various temperatures. The calculated ionic conductivity data are presented in Table 1. As shown in Figure 2c, analysis indicates that the ionic conductivity of the electrolyte membranes with different compositions gradually increases with rising temperature. As clearly observed in Figure 2c, the electrolyte membranes incorporating P(VDF-HFP) exhibit significantly higher ionic conductivities than the pure PEO across all tested temperatures. With increasing P(VDF-HFP) content, the ionic conductivity of the solid electrolyte membranes first increases and then decreases, with the 30%P(VDF-HFP)/70%PEO blend membrane demonstrating the highest ionic conductivity. This indicates that blending P(VDF-HFP) with PEO optimizes lithium ion transport pathways and accelerates lithium ion migration. The enhanced ionic conductivity in P(VDF-HFP)-containing electrolytes is attributed to two key factors: (1) the presence of C-F bonds in P(VDF-HFP), which effectively facilitate the dissociation of lithium salts; and (2) the amorphous regions formed by PEO/P(VDF-HFP) blending, which enable rapid lithium ion migration.

To better evaluate the electrochemical stability window of the polymer solid electrolytes, LSV was performed, with the curves shown in Figure 2d. The decomposition voltage of the pure PEO electrolyte membrane is 4.6 V, whereas that of the 30%-P(VDF-HFP)-containing electrolyte membrane reaches 4.9 V. This phenomenon arises because higher ionic conductivity helps reduce lithium-ion accumulation at the electrode/electrolyte interface, thereby decreasing the interface overpotential and improving compatibility between the electrode and electrolyte [34,35]. The lithium-ion transference number (t_Li_^+^) is an indicator of lithium-ion transport efficiency in electrolytes and a critical parameter for evaluating solid electrolyte performance. A higher t_Li_^+^ signifies better lithium-ion transport efficiency within the electrolyte. DC polarization tests were conducted on Li//Li symmetric cells assembled with pure PEO and the 30%-P(VDF-HFP)/70%-PEO electrolyte (exhibiting the highest ionic conductivity). EIS measurements were performed before and after DC polarization, with the resulting i-t curves and AC impedance spectra presented in Figure 2e and Figure 2f, respectively. As presented in Table 2, the lithium-ion transference number of pure PEO was 0.22, whereas that of the 30%-P(VDF-HFP)/70%-PEO electrolyte reached 0.45, significantly higher than the former. This indicates that the blending P(VDF-HFP) with PEO promotes the dissociation of lithium salts and increases free lithium ion concentration. Combining these results with previous characterization data reveals that the blending modification reduces crystallinity, facilitating lithium-ion migration and thereby enhancing ionic conductivity.

We also measured the ionic conductivity of the polymer solid-state electrolyte prepared from 30%PVDF-HFP and 70%PEO. Its ionic conductivity was 8.89 × 10^−3^ S cm^−1^ at 25 °C and 3.71 × 10^−2^ S cm^−1^ at 60 °C. In contrast, that of the battery assembled with F-PEO/LiTFSI was 3.32 × 10^−4^ S cm^−1^ at 60 °C [36]; the battery assembled with PEO/NaClO_4_/Nano-SiO_2_ had an ionic conductivity of 1.18 × 10^−6^ S cm^−1^ at 60 °C [37]; the battery assembled with PEO-IL/LiTFSI/Ga-LLZO had an ionic conductivity of 5.7 × 10^−4^ S cm^−1^ at 25 °C [38]; and the battery assembled with PEO/PLA/LiClO_4_/MMT had an ionic conductivity of 1.05 × 10^−5^ S cm^−1^ at 25 °C [39]; the ionic conductivity of the battery assembled with PEO/LiTFSI/SBA-LiIL is 4.3 × 10^−4^ S cm^−1^ at 60 °C [40]; the ionic conductivity of the battery assembled with PEO/LiTFSI/PI is 4.2 × 10^−4^ S cm^−1^ at 60 °C [41]. This shows that lithium ions have a more excellent transport efficiency in the battery assembled with 30%PVDF-HFP 70%PEO. The comparison data of their ionic conductivities are shown in Table 3.

**Figure 3:**
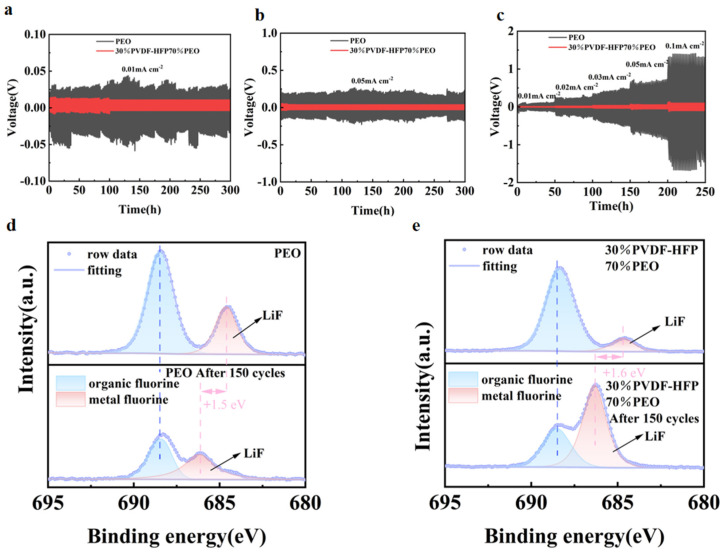
(**a**) Cycling curves of Li/PEO/Li and Li/30%P(VDF-HFP)70%PEO/Li symmetric cells at a current density of 0.01 mA cm^−2^, (**b**) cycling curves of Li/PEO/Li and Li/30%P(VDF-HFP)70%PEO/Li symmetric cells at a current density of 0.05 mA cm^−2^, (**c**) cycling curves of Li/PEO/Li and Li/30%P(VDF-HFP)70%PEO/Li symmetric cells at various current densities, (**d**) XPS of Li/PEO/Li symmetric cells before and after cycling, (**e**) XPS of Li/30%P(VDF-HFP)70%PEO/Li symmetric cells before and after cycling.

To investigate the dynamic stability of the electrolyte membranes on lithium metal anodes, lithium plating-stripping experiments were conducted on Li//Li symmetric cells assembled with PEO and 30%P(VDF-HFP)/70%PEO composite electrolytes. As shown in Figure 3a,b, under current densities of 0.01 mA cm^−2^ and 0.05 mA cm^−2^, the 30%P(VDF-HFP)/70%PEO-based Li//Li symmetric cell exhibits lower polarization voltage compared with the pure PEO electrolyte membrane. This indicates superior stability and lower interface impedance during charge-discharge processes. The long-cycle polarization curves of the composite electrolyte system remained stable, suggesting no fluctuations caused by micro-lithium dendrites piercing the electrolyte membrane. This demonstrates its ability to suppress lithium dendrite growth to a certain extent, effectively improving the electrochemical performance of the battery. As shown in Figure 3c, when the current density was gradually increased from 0.01 mA cm^−2^ to 0.1 mA cm^−2^, the 30%P(VDF-HFP)70%PEO composite electrolyte consistently exhibited much lower polarization voltage compared to pure PEO at each current density, demonstrating excellent rate performance. These test results are attributed to the addition of P(VDF-HFP), which promotes the dissociation of LiTFSI, thereby generating more “LiF” in the electrolyte [42,43]. Through XPS analysis of Li//Li symmetric cells assembled with PEO and 30%P(VDF-HFP)/70%PEO electrolytes, as shown in Figure 3d,e, it can be observed that the LiF content in the PEO electrolyte membrane decreases before and after cycling, whereas the electrolyte membrane containing 30%P(VDF-HFP) generates more LiF during cycling. These LiF species coat the solid-state electrolyte membrane, reducing its contact and reaction with lithium metal. This significantly enhances the stability of the electrolyte membrane and inhibits the growth of lithium dendrites.

**Figure 4:**
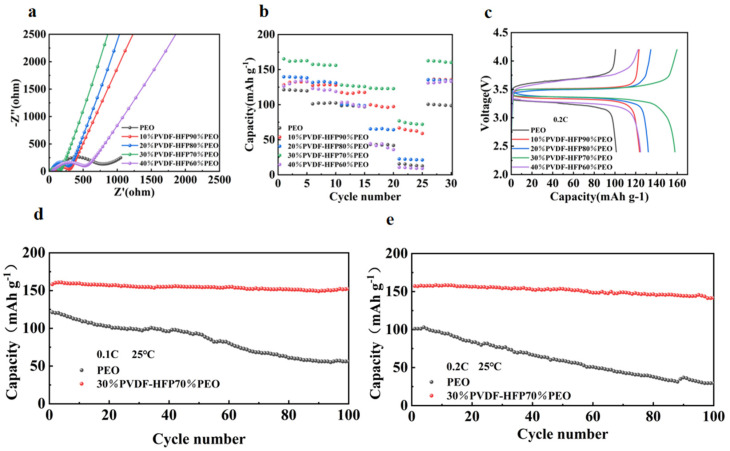
(**a**) EIS curves, (**b**) rate performance, (**c**) initial charge-discharge curves at 0.2C, (**d**) cycling performance at 0.1C, (**e**) cycling performance at 0.2C.

To investigate the interface compatibility between electrolytes and electrodes, solid-state batteries were assembled using pure PEO and composite electrolytes composed of P(VDF-HFP) and PEO at various ratios. EIS measurements were performed on PEO, 10%P(VDF-HFP)/90%PEO, 20%P(VDF-HFP)/80%PEO, 30%P(VDF-HFP)/70%PEO, and 40%P(VDF-HFP)/60%PEO electrolytes. As shown in Figure 4a, the cell assembled with 30%P(VDF-HFP)/70%PEO electrolyte exhibits the smallest charge transfer resistance (Rct), indicating superior charge transfer capability. As shown in Figure 4b, when the current density was increased from 0.1C to 2C, the solid electrolyte composed of 30%P(VDF-HFP)/70%PEO blend exhibits reversible capacities of 165.27 mAh g^−1^, 157.42 mAh g^−1^, 128.02 mAh g^−1^, 123.59 mAh g^−1^, and 76.8 mAh g^−1^ at 0.1C, 0.2C, 0.5C, 1C, and 2C respectively, which are all significantly higher than those of other samples. Furthermore, when the current density was returned to 0.1C, the cell retained a high specific capacity of 162.57 mAh g^−1^, indicating that the polymer solid electrolyte with appropriate P(VDF-HFP) addition to PEO demonstrated excellent reversible performance.

As shown in the initial charge-discharge curves of Figure 4c, the 30%P(VDF-HFP)/70%PEO composite electrolyte delivers the highest specific capacity at a current density of 0.2C. Figure 4d presents the cycling performance of PEO and 30%P(VDF-HFP)/70%PEO electrolytes at 0.1C and 0.2C. At 0.1C, the 30%P(VDF-HFP)/70%PEO electrolyte maintains a higher specific capacity than pure PEO, retaining 152.29 mAh g^−1^ after 100 cycles, whereas the PEO electrolyte only retains 56.09 mAh g^−1^ indicating poor cycle stability. As shown in Figure 4e, the 30%P(VDF-HFP)/70%PEO composite electrolyte exhibits higher specific capacity than pure PEO at a current density of 0.2C, maintaining a high specific capacity of 141.59 mAh g^−1^ after 100 cycles. These results indicate that the addition of appropriate amounts of P(VDF-HFP) to PEO can suppress the lithium dendrite growth, preventing structural damage caused by dendrites, and thereby endows the assembled batteries with excellent cycle performance.

**Figure 5:**
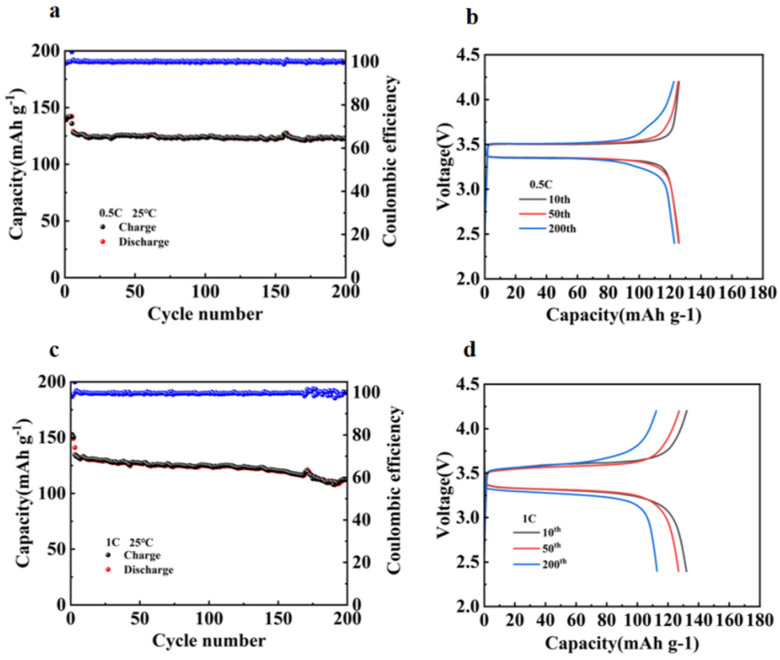
(**a**) Long-term cycling performance of 30%P(VDF-HFP)/70%PEO at 0.5C, (**b**) charge-discharge curves of 30%P(VDF-HFP)/70%PEO at 0.5C, (**c**) long-term cycling performance of 30%P(VDF-HFP)/70%PEO at 1C, (**d**) charge-discharge curves of 30%P(VDF-HFP)/70%PEO at 1C.

As shown in the cycling performance at 0.5C in Figure 5a, the cell assembled with 30%P(VDF-HFP)/70%PEO blend maintained a specific capacity of 122.39 mAh g^−1^ after 200 cycles, demonstrating remarkably high capacity retention. This finding was further validated by the charge-discharge curves in Figure 5b, where no significant polarization changes were observed after 0, 50, and 200 cycles. As shown in Figure 5c, the cell assembled with 30%P(VDF-HFP)/70%PEO blend also demonstrated excellent cycle stability at a high current density of 1C, retaining a reversible specific capacity of 112.77 mAh g^−1^ after 200 cycles. The curves in Figure 5d further validated this result by showing consistent capacity retention. These favorable outcomes can be attributed to the addition of appropriate amounts of P(VDF-HFP), which effectively suppresses lithium dendrite growth and endows the assembled batteries with outstanding cycle performance.

## 3. Experimental

### 3.1. Materials

PEO(average Mw~1,000,000), P(VDF-HFP)(average Mw~455,000), anhydrous acetonitrile, LiTFSI (lithium bis(trifluoromethanesulfonyl)imide), LiFePO_4_, super P, PVDF, and NMP were purchased from Shanghai Aladdin Biochemical Technology Co., Ltd. (Shanghai, China).

### 3.2. Blending of PEO and P(VDF-HFP)

**Figure 6:**
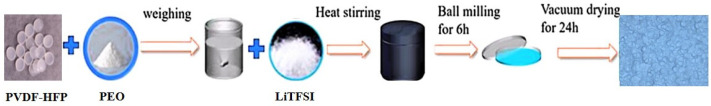
Schematic illustration of the preparation process for PEO/P(VDF-HFP) blend solid electrolyte.

PEO and P(VDF-HFP) were blended at different ratios. Appropriate amounts of lithium salt LiTFSI were added to the blends containing varying proportions of PEO and P(VDF-HFP), followed by the addition of anhydrous acetonitrile. The mixture was then heated at a constant temperature in a beaker until it dissolved into a viscous state. This viscous solution was transferred to a ball-milling jar and ball-milled for 6 h. The resulting slurry was poured into a glass petri dish. The glass petri dish was first placed in a vacuum environment for about 2 h to remove air bubbles and dried in a vacuum oven at 60 °C for 24 h to form freestanding films, yielding polymer solid-state electrolytes with different compositions. The preparation process is schematically illustrated in Figure 6.

### 3.3. Preparation of the Cathode

To prepare the cathode electrode, LiFePO_4_, super P, and PVDF (polyvinylidene fluoride) were mixed at a weight ratio of 8:1:1. An appropriate amount of 1-methyl-2-pyrrolidinone (NMP) solvent was then added, and the mixture was transferred to a ball-milling jar and stirred for 12 h. The resulting slurry was uniformly coated onto aluminum foil, which was subsequently dried in a vacuum oven at 60 °C for 12 h. Finally, the aluminum foil was cut into small circular pieces with a diameter of 13 mm, yielding electrodes with a loading of approximately 3 mg cm^−2^. All CR2016 battery assemblies were conducted in an argon-filled glovebox.

### 3.4. Characterization and Electrochemical Testing

The surface morphology and microstructure of the experimental materials were characterized using a laser confocal microscope. The XRD tests we conducted were carried out within the 2θ range of 10° to 30° C. Thermogravimetric analysis (TGA) was performed under a nitrogen atmosphere at a heating rate of 10 °C/min within the temperature range of 25 °C to 800 °C.

The stainless steel (SS)/solid electrolyte/Li cells were assembled and tested using linear sweep voltammetry (LSV) on an electrochemical workstation to evaluate the electrochemical stability of the solid electrolytes. The scan rate was set at 1 mV s^−1^, with a voltage scan window ranging from 2 to 6 V.

The SS/solid electrolyte/SS cells were assembled and subjected to electrochemical impedance spectroscopy (EIS) measurements within a frequency range of 0.1 Hz to 10^5^ Hz with an AC amplitude of 10 mV. The ionic conductivity (σ) of the prepared solid electrolyte membranes was calculated using the following formula:

$$\sigma =\frac{L}{R_{b}S}$$

Here, σ represents the ionic conductivity of the solid electrolyte (S·cm^−1^), L denotes the thickness of the prepared solid electrolyte membrane during the experiment (cm), R_b_ refers to the impedance value of the solid electrolyte membrane obtained via electrochemical impedance spectroscopy (Ω), and S indicates the area of the SS electrode used (cm^2^).

Following the temperature-dependent impedance measurements, linear fitting of σ at different temperatures was performed using the Arrhenius equation, with the calculation formula expressed as follows:

$$\sigma =\sigma_{0}\exp(-\frac{Ea}{KT})$$

Here, σ_0_ represents the pre-exponential factor, K is the Boltzmann constant (8.61 × 10^−5^ eV·K^−1^), T denotes the thermodynamic temperature (also known as absolute temperature, calculated by adding 273 to the Celsius temperature, with units of K), and E_a_ corresponds to the apparent activation energy for ionic transport in the solid electrolyte.

The lithium-ion transference number (t_Li_^+^) can be calculated from the AC impedance spectra and DC polarization curves of Li/solid electrolyte/Li cells recorded on an electrochemical workstation. The formula for calculating the lithium-ion transference number (t_Li_^+^) is as follows:

$${t_{Li}}^{+}=\frac{I_{S}(\Delta V-I_{0}R_{0})}{I_{0}(\Delta V-I_{S}R_{S})}$$

ΔV represents the bias voltage applied during DC polarization. I_0_ and I_S_ denote the initial and steady-state current values during DC polarization, respectively; R_0_ and R_S_ represent the interface impedance values of the battery before and after DC polarization.

Cycling tests were conducted on Li/PEO/Li and Li/30%-P(VDF-HFP)-70%-PEO/Li cells at various current densities using a testing system to evaluate their lithium plating-stripping performance under different currents. Additionally, electrochemical performance tests were performed on LFP/solid electrolyte/Li cells within a voltage range of 2.4–4.2 V.

## 4. Conclusions

In this study, a novel polymer solid electrolyte was prepared via blending PEO with P(VDF-HFP), and their electrochemical performance was systematically investigated. Experimental results demonstrated that blending PEO with P(VDF-HFP) significantly improved the overall performance of the solid electrolyte, notably in terms of ionic conductivity, electrochemical stability, interface compatibility, and thermal stability. The results indicated that by optimizing the P(VDF-HFP) content, the overall performance of the solid electrolyte can be effectively enhanced, providing strong support for next-generation batteries with high safety and high energy density.

## Figures and Tables

**Table 1:** Ionic conductivities of various electrolytes at different temperatures (units of ionic conductivity: S cm^−1^).

Temperature (°C)	25 °C	30 °C	35 °C	40 °C	45 °C	50 °C	55 °C	60 °C
Electrolyte								
PEO	3.86 × 10^−4^	4.43 × 10^−4^	6.77 × 10^−4^	8.13 × 10^−4^	1.46 × 10^−3^	1.69 × 10^−3^	2.42 × 10^−3^	3.18 × 10^−3^
10%P(VDF-HFP)90%PEO	1.64 × 10^−3^	1.93 × 10^−3^	2.23 × 10^−3^	3.25 × 10^−3^	3.85 × 10^−3^	4.77 × 10^−3^	6.53 × 10^−3^	7.87 × 10^−3^
20%P(VDF-HFP)80%PEO	1.92 × 10^−3^	2.31 × 10^−3^	2.87 × 10^−3^	3.79 × 10^−3^	4.84 × 10^−3^	5.76 × 10^−3^	6.79 × 10^−3^	7.95 × 10^−3^
30%P(VDF-HFP)70%PEO	8.89 × 10^−3^	1.68 × 10^−2^	2.35 × 10^−2^	2.56 × 10^−2^	2.76 × 10^−2^	2.98 × 10^−2^	3.23 × 10^−2^	3.71 × 10^−2^
40%P(VDF-HFP)60%PEO	9.01 × 10^−4^	1.16 × 10^−3^	1.37 × 10^−3^	2.79 × 10^−3^	3.53 × 10^−3^	4.00 × 10^−3^	4.52 × 10^−3^	4.70 × 10^−3^

**Table 2:** Lithium-ion transference numbers of PEO and 30%P(VDF-HFP)/70%PEO.

Polymer Solid Electrolyte	ΔV (V)	Ι0 (μA)	Ιs (μA)	R0 (Ω)	Rs (Ω)	t_Li_^+^
PEO	0.01	36.60	12.90	150.64	221.37	0.22
30%P(VDF-HFP)70%PEO	0.01	40.75	24.36	124.03	139.21	0.45

**Table 3:** A comparison of the ionic conductivity of 30%PEO/70%P(VDF-HFP) with data from other literature.

	Solid Electrolyte	T (°C)	Ionic Conductivity (S cm^−1^)	Ref.
1	F-PEO/LiTFSI	60	3.32 × 10^−4^	[36]
2	PEO/NaClO4/Nano-SiO2	60	1.18 × 10^−6^	[37]
3	PEO-IL/LiTFSI/Ga-LLZO	25	5.7 × 10^−4^	[38]
4	PEO/PLA/LiClO4/MMT	25	1.05 × 10^−5^	[39]
5	PEO/LiTFSI/SBA-LiIL	60	4.3 × 10^−4^	[40]
6	PEO/LiTFSI/PI	60	4.2 × 10^−4^	[41]
7	70%PEO/LiTFSI/30%P(VDF-HFP)	25 60	8.89 × 10^−3^ 3.71 × 10^−2^	This paper

## Data Availability

The original contributions presented in this study are included in the article.
